# The Optimal Regimen for the Treatment of Temporomandibular Joint Injury Using Low-Intensity Pulsed Ultrasound in Rats with Chronic Sleep Deprivation

**DOI:** 10.1155/2020/5468173

**Published:** 2020-05-01

**Authors:** Chao Liang, Tao Yang, Gaoyi Wu, Jun Li, Wei Geng

**Affiliations:** ^1^Department of Dental Implant Center, Beijing Stomatological Hospital, School of Stomatology, Capital Medical University, Beijing 100050, China; ^2^Department of Stomatology, Jinan Military General Hospital, Jinan 250000, China; ^3^Beijing Key Laboratory of Tooth Regeneration and Function Reconstruction, School of Stomatology, Capital Medical University, Beijing 100050, China

## Abstract

Low-intensity pulsed ultrasound (LIPUS) is an emerging physical therapy for the treatment of early temporomandibular joint injury and has a good effect on promoting cartilage and subchondral bone tissue repair. However, the best LIPUS intensity and treatment duration remain unclear. This study is aimed at observing the preventive and therapeutic effects of different modes of LIPUS and at identifying the optimal LIPUS treatment regimen for temporomandibular joint injury. In the present study, rat models of temporomandibular joint injury were established using a chronic sleep deprivation (CSD) method, and the effect of LIPUS as intensities of 30, 45, and 60 mW/cm^2^ was observed at 7, 14, and 21 days. After CSD, the condylar cartilage of the rats demonstrated variable degrees of surface roughening, collagen fiber disarrangement or even partial exfoliation, decreased proteoglycan synthesis and cartilage thickness, decreased chondrocyte proliferation, decreased type 2 collagen (COL-2) expression, and increased matrix metalloproteinase- (MMP-) 3 expression at all three time points. When the rats with CSD received different intensities of LIPUS treatment, the pathological changes were alleviated to various extents. The groups receiving 45 mW/cm^2^ LIPUS showed the most significant relief of cartilage damage, and this significant effect was observed on days 14 and 21. These results demonstrated that LIPUS can effectively inhibit CSD-induced condylar cartilage damage in rats, and LIPUS treatment at an intensity of 45 mW/cm^2^ for at least 2 weeks is the optimal regimen for temporomandibular joint injury.

## 1. Introduction

Temporomandibular joint disorder (TMD) is a common and highly prevalent disease of the oral and maxillofacial region. The main pathological changes include articular disc and condylar cartilage inflammation, degenerative changes, condylar surface injury, and cartilage vascularization [1, 2]. TMD treatments can be divided into noninvasive, minimally invasive, and invasive according to the degree of trauma induced [3]. When developing a treatment plan, the least traumatic option with optimal efficacy is usually preferred [4]. However, the current noninvasive treatment for TMD mainly focuses on regulating occlusal disorders or mental factors, and a direct treatment for the injury site is still lacking, which is the main reason for failure to quickly and effectively relieve local TMD symptoms [5]. Therefore, directly and effectively controlling local inflammation and promoting cartilage repair have become urgent problems that must be resolved for TMD treatment.

Low-intensity pulsed ultrasound (LIPUS) is a noninvasive local treatment method that acts on the affected area using pulsed ultrasound with an output intensity of less than 100 mW/cm^2^ [6]. Many biological effects can be induced through sound waves, for example, increased protein synthesis, enhanced cellular proliferation, and increased second messenger Ca^2+^ uptake, which can then engender therapeutic effects. LIPUS is a safe treatment method characterized by good targeting, minimal heat effects, and no harm to adjacent tissues [7]. LIPUS has demonstrated significant effects on the repair of bone injury and nerve damage and the promotion of microcirculation in soft tissue [8–10]. Moreover, studies have found that LIPUS can stimulate rat chondrocyte proliferation, which also has a certain therapeutic effect on articular cartilage injury [11, 12], and rats are an ideal model to observe the growth and injury of the mandibular condyle [13].

However, LIPUS treatment involves many parameters, and differences in intensity and duration will affect the biological effects to a certain extent. At present, most LIPUS-related studies on cartilage refer to the ultrasound parameters for bone fracture treatment, and the ultrasound modes that are most beneficial for cartilage repair are still unclear. Considering the above problems, this study is aimed at evaluating the preventive and treatment effects of different LIPUS intensities for different durations on temporomandibular joint injury in rats and at identifying the optimal regimen, providing both an experimental basis for further research on the molecular mechanism of LIPUS treatment and a theoretical basis for the clinical application of LIPUS in TMD treatment.

## 2. Materials and Methods

### 2.1. Experimental Animals

All animal experiments performed in this study were reviewed and approved by the Animal Ethics Committee of Capital Medical University (Beijing, China) in strict accordance with NIH guidelines (permit number: KQYY-201610-001). A total of 150 8-week-old male-specific pathogen-free (SPF) Wistar rats weighing 200 ± 20 gwere purchased from the Sipeifu Experimental Animal Center (Beijing, China). The rats were adaptively housed for 1 week before the experiment, fed with a normal diet, and maintained under a 12 h/12 h light/dark cycle and a temperature of 20 ± 3°C.

### 2.2. Experimental Grouping

The rats were randomly divided into the following five groups with 30 rats per group: the control (CON), chronic sleep deprivation (CSD), CSD+LIPUS 30 mW/cm^2^ intervention (LIPUS 30), CSD+LIPUS 45 mW/cm^2^ intervention (LIPUS 45), and CSD+LIPUS 60 mW/cm^2^ intervention (LIPUS 60) groups. Each group was further randomly divided into three subgroups according to the observation time (7, 14, and 21 days), with 10 rats per subgroup.

### 2.3. CSD and LIPUS Treatment

Similar to our previous study [14], the rats were subjected to CSD using the improved multiplatform method to establish the model of temporomandibular joint injury. The rats in the CSD and LIPUS 30, 45, and 60 groups were placed on several platforms in a sleep deprivation water tank. When a rat entered the rapid eye movement sleep period, whole-body muscle tension dropped, the head touched the water surface, and the rat woke up, thereby achieving sleep deprivation. The rats were deprived of sleep for 22 h daily from 13:00 pm to 11:00 am the next day and allowed to sleep for 2 h as a buffer. The CON group rats were placed in a sleep deprivation water tank with a grid to prevent the rats from falling into the water.

The LIPUS 30, 45, and 60 group rats received the LIPUS intervention at the corresponding intensity for 20 min daily [15] under isoflurane gas anesthesia while simultaneously undergoing CSD. The bilateral temporomandibular joint intervention was performed using the OSTEOTRON IV ultrasound equipment (Ito Ultrawave Co., Ltd., Tokyo, Japan), the ultrasound intensity of which includes three levels (30, 45, and 60 mW/cm^2^): the ultrasound frequency was 1.0 MHz, the pulse width was 200 *μ*s, and the repetition rate was 1 kHz.

### 2.4. Hematoxylin-Eosin (HE) and Toluidine Blue (TB) Staining

All rats were dissected after sacrifice to collect bilateral temporomandibular joint specimens. Left joint specimens were fixed in 4% paraformaldehyde for 48 h, decalcified in 10% EDTA for 2 months, embedded in paraffin, sliced to 5 *μ*m thickness along the coronal plane, and stained with HE and TB (Solarbio, Beijing, China). Using a blind method, two researchers recorded scores for joint pathological changes using the modified Mankin scoring system [16]. The Mankin score ranges from 4 to 16 points, and a lower score indicates less severe pathological changes. In addition, the distance from the surface of the external fibrous layer to the tidemark was measured in the central section of the condyle using Image-Pro Plus 6.0 software. The mean of three different measurement points was used to represent the thickness of the condylar cartilage.

### 2.5. Immunofluorescence Assay for Proliferating Cell Nuclear Antigen (PCNA)

The left joint specimen sections were dewaxed. The antigen was restored using 0.01 M sodium citrate in a 95°C water bath, and endogenous enzyme activity was inactivated with 3% H_2_O_2_. The sections were blocked with goat serum and incubated with PCNA primary antibody (1 : 200) (Abclonal, Wuhan, China) at 4°C overnight. The sections were then incubated with a Rhodamine red fluorescent secondary antibody (Abclonal, Wuhan, China) and stained with 4′,6-diamidino-2-phenylindole (DAPI) (Abclonal, Wuhan, China) to visualize the nuclei. Image-Pro Plus 6.0 software was used to calculate the PCNA average optical density (AOD) score.

### 2.6. Immunohistochemistry Detection of Type 2 Collagen (COL-2) and Matrix Metalloproteinase- (MMP-) 3

The left joint specimen sections were processed as described for the immunofluorescence assay up to the incubation step with the primary antibody. The antibody dilution ratio for COL-2 was 1 : 200, and the ratio for MMP-3 was 1 : 500 (Abcam, Cambridge, UK). The sections were then incubated with a biotin-labeled anti-rabbit secondary antibody (ZSGB-BIO, Beijing, China), stained with 3,3′-diaminobenzidine (DAB) solution (ZSGB-BIO, Beijing, China), counterstained with hematoxylin to visualize the nuclei, and sealed with neutral gum. Image-Pro Plus 6.0 software was used to calculate the AOD score of the positive product.

### 2.7. Reverse Transcription Polymerase Chain Reaction (RT-PCR)

Rat right joint specimens were prepared to acquire the condylar cartilage. After flash-freezing in liquid nitrogen, the specimens were ground in TRIzol reagent (Invitrogen, Carlsbad, USA). Total RNA was extracted using a tissue RNA extraction kit (CWBIO, Beijing, China). Then, cDNA was reverse transcribed using a PrimeScript RT Reagent Kit and gDNA Eraser (Takara, Shiga, Japan), and real-time PCR was accomplished using a SYBR Premix Ex Taq II kit (Takara, Shiga, Japan). The PCNA, COL-2, and MMP-3 primer sequences are shown in Table 1.

### 2.8. Statistical Analysis

SPSS 21.0 software was employed for statistical analysis. All tests were performed in triplicate and are presented as the mean ± SD. Quantitative data were analyzed using one-way analysis of variance (ANOVA) for multiple comparisons. Comparisons between two groups were tested using Student's *t*-test. The level of significance was defined according to two *P* values (^∗^*P* < 0.05, ^∗∗^*P* < 0.01).

## 3. Results

### 3.1. Histological Changes and Mankin Scores

**Figure 1:**
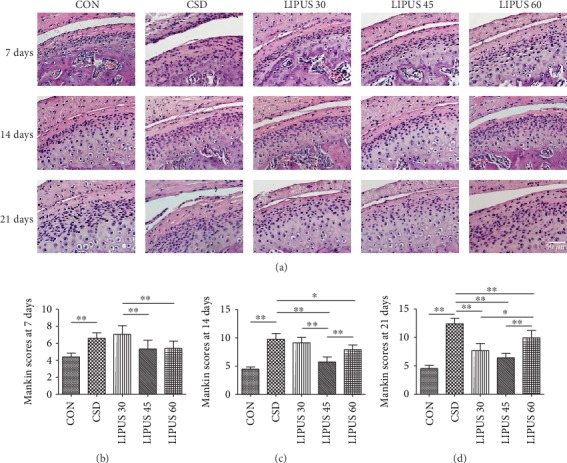
HE staining and Mankin scores of the condylar cartilage. (a) Compared with the CON group, the cartilage surface of the CSD group was rough, the chondrocytes were irregularly arranged, and partial fiber exfoliation was visible at day 21. LIPUS intervention improved the pathological changes in the condylar cartilage, especially in the LIPUS 45 group after 14 days. (b–d) The Mankin scoring results showed that the CSD group's score was significantly higher than the CON group's score at three time points. When LIPUS intervention was administered for 14 days or longer, the score of the LIPUS 45 group was significantly lower than that of the CSD group, and the declining trend was more significant than those of the LIPUS 30 and 60 groups. The data are recorded as the mean ± SD. ^∗^*P* < 0.05; ^∗∗^*P* < 0.01.

**Figure 2:**
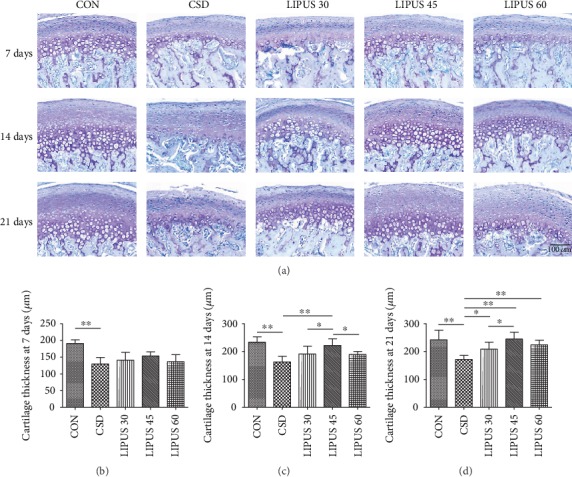
Toluidine blue staining and thickness measurement in the condylar cartilage. (a) Toluidine blue staining showed that proteoglycan synthesis in the cartilage was decreased in the CSD group compared with the CON group and was increased at 14 and 21 days of LIPUS treatment, especially in the LIPUS 45 group. (b–d) The measurement results showed significantly reduced cartilage thickness in the CSD group, and the thickness was significantly rescued in the LIPUS 45 group at day 14 and in all three LIPUS groups at day 21. The data are recorded as the mean ± SD. ^∗^*P* < 0.05; ^∗∗^*P* < 0.01.

HE staining showed no abnormalities of the condylar cartilage in the CON group. However, in the CSD group, the condylar cartilage surface showed increased roughness, and the chondrocytes were arranged in a disorderly manner. The continuity of the fibrous layer was significantly damaged at day 21, and exfoliated fibers were even present in the lower joint cavity. When treated with different LIPUS intensities, the pathological changes in the condylar cartilage were improved to various extents, and the improvements began to be significant starting from 14 days. In the LIPUS 45 group, the surface of the fibrous layer was smooth, the number of chondrocytes increased significantly, and the regular arrangement of the chondrocytes was restored, reflecting the most significant improvement in pathological changes (Figure 1(a)). The TB staining showed that the proteoglycan distribution in the condylar cartilage was decreased in the CSD groups compared with the CON groups. However, when receiving LIPUS treatment for 14 days or longer, the proteoglycan distribution was rescued to various degrees, and the increased level in the LIPUS 45 groups was more obvious (Figure 2(a)).

The Mankin scores were significantly increased starting from day 7 of CSD treatment (*P* < 0.01) (Figures 1(b), 1(c), and 1(d)). At day 14 of the LIPUS intervention, the score of the LIPUS 45 group was significantly decreased compared with that of the CSD group and exhibited a more significant downward trend than those of the LIPUS 30 and 60 groups (*P* < 0.01) (Figure 1(c)). At day 21, all LIPUS groups exhibited a good therapeutic effect, although the LIPUS 45 group still demonstrated the most obvious effect (Figure 1(d)).

The thickness measurement results revealed that the cartilage thickness in the CSD group was significantly reduced from day 7 (Figure 2(b)). Starting from day 14, the cartilage thickness was significantly increased in the LIPUS 45 group (Figure 2(c)). At day 21, the three LIPUS groups all showed increased cartilage thickness, and the 45 mW/cm^2^ and 60 mW/cm^2^ LIPUS intensities were found to have more significant rescue effects (*P* < 0.01) (Figure 2(d)).

### 3.2. Fluorescence Expression and Quantitative Analysis of PCNA in Condylar Cartilage

**Figure 3:**
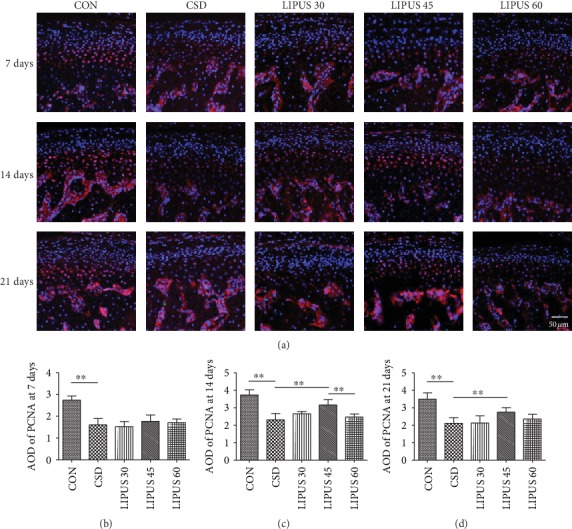
Immunofluorescence staining and quantitative analysis of PCNA in the condylar cartilage. (a) PCNA expression in the condylar chondrocytes of the CSD group rats was significantly decreased compared with that in the CON group but was significantly increased when the rats simultaneously received LIPUS treatment for 14 days or longer. The most significant effect was observed in the LIPUS 45 group. (b–d) The AOD of PCNA was significantly increased in the LIPUS 45 group at days 14 and 21. The data are recorded as the mean ± SD. ^∗^*P* < 0.05; ^∗∗^*P* < 0.01.

Red fluorescence indicated PCNA expression in the nuclei of chondrocytes in each group, and DAPI showed blue fluorescence. CSD treatment significantly decreased PCNA expression in chondrocytes, whereas the LIPUS intervention in each experimental group restored PCNA expression to different degrees (Figure 3(a)). The effects of LIPUS were not obvious at day 7 (Figure 3(b)), but at day 14 and day 21, PCNA expression in the LIPUS 45 group significantly increased compared with that in the CSD group (*P* < 0.01) (Figures 3(c) and 3(d)).

### 3.3. COL-2 Expression and Quantitative Analysis in Condylar Cartilage

**Figure 4:**
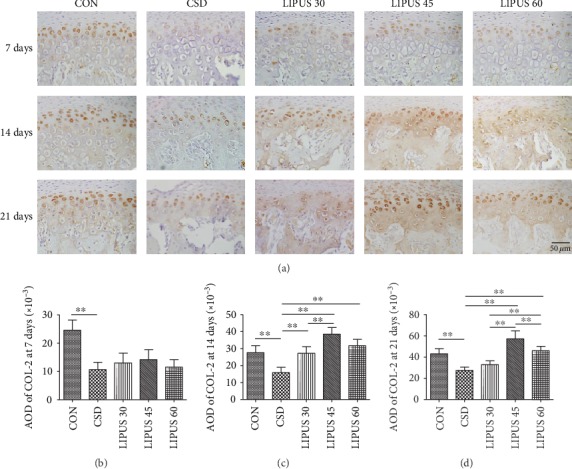
Immunohistochemical staining and quantitative analysis of COL-2 in the condylar cartilage. (a) COL-2 expression was significantly decreased at all three time points in the CSD groups and significantly increased starting at day 14 of LIPUS treatment. The increase was most significant in the LIPUS 45 group. (b–d) Corresponding to the microscopic images, the AOD of COL-2 was significantly increased at days 14 and 21 in the LIPUS groups, and the most significant effect was observed at an intensity of 45 mW/cm^2^. The data are recorded as the mean ± SD. ^∗^*P* < 0.05; ^∗∗^*P* < 0.01.

COL-2 is mainly expressed in the cytoplasm of chondrocytes and the surrounding cartilage matrix. CSD treatment significantly reduced COL-2 expression in condylar cartilage. Starting at day 14 of the LIPUS intervention, COL-2 expression began to significantly increase and even reached a level higher than the normal value. The increasing level was more significant in the LIPUS 45 group than those in the other two LIPUS groups (Figure 4(a)). The AOD of COL-2 in the CSD group was significantly lower than that in the CON group at the three time points (*P* < 0.01). The effect of LIPUS was not obvious at day 7 (Figure 4(b)), but at day 14, COL-2 expression began to significantly increase in all three LIPUS groups (*P* < 0.01), with the LIPUS 45 group showing the most significant effect (Figure 4(c)). At day 21, LIPUS at an intensity of 45 mW/cm^2^ was also found to be more advantageous (*P* < 0.01) (Figure 4(d)).

### 3.4. MMP-3 Expression and Quantitative Analysis in Condylar Cartilage

**Figure 5:**
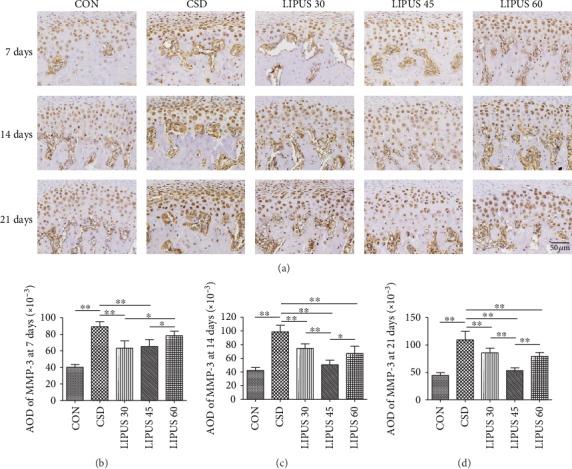
Immunohistochemical staining and quantitative analysis of MMP-3 in the condylar cartilage. (a) Beginning from day 7, MMP-3 expression in the CSD group was significantly higher than that in the CON group, and the expression decreased to various degrees when the rats with CSD received LIPUS treatment. The change in the LIPUS 45 group was most significant. (b–d) The AOD of MMP-3 was significantly decreased in the LIPUS 30 and 45 groups at day 7 and was significantly decreased in all three LIPUS groups at days 14 and 21. Again, the most significant effect was observed at an intensity of 45 mW/cm^2^. The data are recorded as the mean ± SD. ^∗^*P* < 0.05; ^∗∗^*P* < 0.01.

MMP-3 is mainly expressed in the cytoplasm of chondrocytes. MMP-3 expression was strongly positive in the CSD groups, but the intensity was significantly reduced in the LIPUS groups, with the most significant changes in the LIPUS 45 group at days 14 and 21 (Figure 5(a)). The AOD of MMP-3 in the CSD group at each time point was significantly higher than that in the CON group (*P* < 0.01). At day 7, the AOD was decreased in the LIPUS 30 and LIPUS 45 groups (*P* < 0.01) (Figure 5(b)). At day 14, all three LIPUS groups showed significant changes, and the decreased MMP-3 level in the LIPUS 45 group was more significant than those in the other two LIPUS groups (*P* < 0.01) (Figure 5(c)). The results at day 21 were similar to those at day 14 (Figure 5(d)).

### 3.5. PCNA, COL-2, and MMP-3 mRNA Expression Levels in Condylar Cartilage

**Figure 6:**
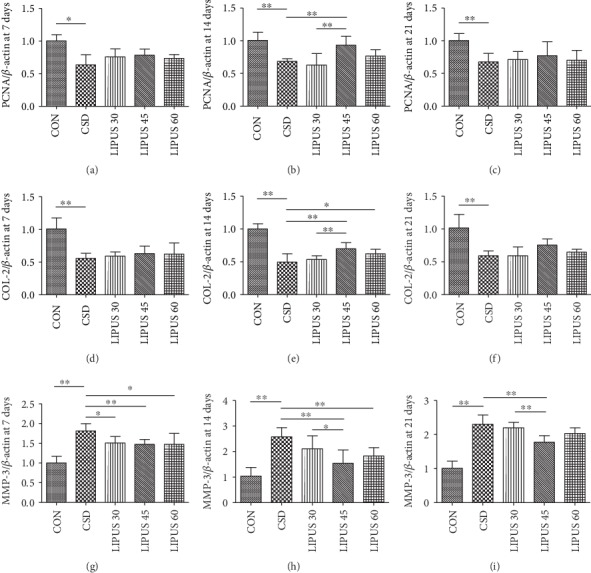
Comparison of PCNA, COL-2, and MMP-3 mRNA expression in the condylar cartilage. (a–c) PCNA mRNA expression was significantly decreased after CSD. When receiving LIPUS treatment, the expression level in the LIPUS 45 group was significantly increased at day 14. (d–f) COL-2 mRNA expression was also significantly decreased after CSD and significantly increased in the LIPUS 45 group at day 14. (g–i) MMP-3 mRNA expression was significantly increased after CSD and decreased starting from day 7 of LIPUS treatment. The decrease was most significant in the LIPUS 45 group at all 3 time points. Data are recorded as the mean ± standard deviation. ^∗^*P* < 0.05; ^∗∗^*P* < 0.01.

**Figure 7:**
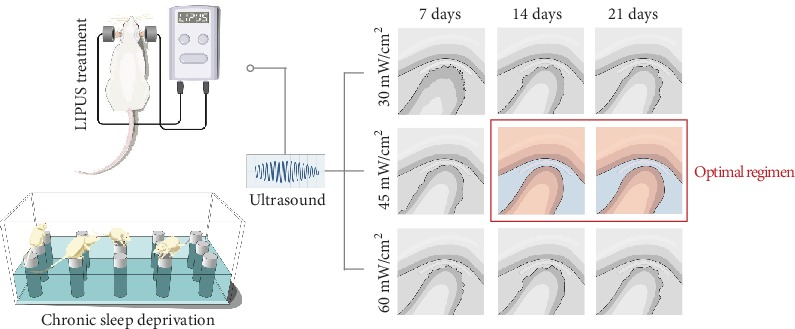
Schematic model for the findings of this study. LIPUS treatment at an intensity of 45 mW/cm^2^ for at least 2 weeks is the optimal regimen for the treatment of temporomandibular joint injury induced by CSD in rats.

Although the changes in PCNA mRNA expression at days 7 and 21 were not significant (Figures 6(a) and 6(c)), the PCNA level in the LIPUS 45 group at day 14 was significantly higher than that in the CSD group (*P* < 0.01) (Figure 6(b)). The changes in COL-2 mRNA expression at days 7 and 21 were not significant (Figures 6(d) and 6(f)), but at day 14, the COL-2 level in the LIPUS 45 group was significantly increased (*P* < 0.01), and the COL-2 level in the LIPUS 60 group was also different from that in the CSD group (*P* < 0.05) (Figure 6(e)). MMP-3 mRNA expression in the three LIPUS groups was obviously decreased at day 7 (*P* < 0.05), and the difference between the LIPUS 45 and CSD groups was most significant (*P* < 0.01) (Figure 6(g)). At days 14 and 21, the changes in the LIPUS 45 group were even more significant (*P* < 0.01) (Figures 6(h) and 6(i)). All of the above findings confirmed that treatment with LIPUS at an intensity of 45 mW/cm^2^ for 14 days or longer had optimal therapeutic effects (Figure 7).

## 4. Discussion

Our previous study found that LIPUS had a good treatment effect on early temporomandibular joint injury and could stimulate chondrocyte proliferation, accelerate cartilage tissue metabolism, relieve inflammation, and promote cartilage and subchondral bone tissue repair by regulating the MMP-3/TIMP-1 and RANKL/OPG expression ratios in cartilage tissue [17]. However, no clear conclusion has been reached regarding the optimal LIPUS intensity and treatment duration for the prevention and treatment of articular cartilage injury.

Previous studies confirmed that sleep disorders can reduce the body's immunity and caused psychological stress, which play important roles in TMD pathogenesis [18, 19]. Multiple studies by our research group have confirmed that the CSD method can successfully establish a stable rat model of temporomandibular joint injury [14, 17, 18, 20]. In this study, the articular cartilage of rats with CSD showed obvious pathological changes, which is consistent with previous literature reports.

The condylar cartilage consists of four layers: the external fibrous layer, followed by the proliferative, chondrocyte, and calcified cartilage layers [21]. The fibrous layer of young or normal condyles is consecutive and smooth, and chondrocytes in the chondrocyte layer are oriented in an orderly manner. However, in old condyles or under pathological conditions, the surface of the fibrous layer becomes rough, chondrocytes are disordered in the chondrocyte layer, and the number of chondrocytes is significantly reduced or even eliminated [22]. In this study, similar pathological changes as described above were observed in the CSD rat models, and LIPUS intervention could mitigate these changes. The HE and TB staining results showed that LIPUS at the 45 mW/cm^2^ intensity yielded the most obvious therapeutic effects starting from 14 days. Previous studies have shown that reduced chondrocyte proliferation is one obvious pathologic change in condylar cartilage degenerative lesions [23], and the restoration of chondrocyte proliferation could indicate reduced disease progression and joint injury [24]. According to this theoretical basis, the expression of the cellular proliferation-related factor PCNA in the middle zones of rat condylar cartilage tissue was further examined in this study. PCNA is intimately related to DNA synthesis in cells, plays an important role in the initiation of cell proliferation, and is a recognized indicator reflecting the state of cell proliferation [25]. At days 14 and 21 of the LIPUS intervention, the number of PCNA-positive cells was significantly increased, and the most significant effect was observed at the 45 mW/cm^2^ intensity. Based on a comprehensive analysis of the morphology and immunofluorescence results, we preliminarily speculate that LIPUS at a 45 mW/cm^2^ intensity has a better effect on the prevention and treatment of condylar cartilage injury in CSD rats. Moreover, when the intervention is applied for 2 weeks, the treatment effect begins to be definitive.

To further explore how LIPUS inhibits pathological changes, we focused on the expression of COL-2 and MMP-3 in condylar cartilage tissue. Under normal conditions, the cartilage matrix is composed of abundant collagen and proteoglycans [26]. COL-2 accounts for more than 90% of the total collagen and not only maintains the organic shape of cartilage tissue but also constitutes the microenvironment in which the chondrocytes perform their metabolic functions [27]. The COL-2 content directly reflects the metabolic activity and health status of the cartilage [28]. In this study, consistent with the histomorphological results, COL-2 mRNA and protein expression levels were significantly reduced when the rat condylar cartilage was injured, but the levels were restored starting from 2 weeks of LIPUS treatment. LIPUS at a 45 mW/cm^2^ intensity again demonstrated the most significant effect.

MMPs constitute a family of proteolytic enzymes that are widely present in connective tissues and are considered one of the important regulatory systems for physiological reconstruction and pathological destruction of body tissues [29]. Studies have confirmed that MMP-3 is the most important hydrolase, which has a strong ability to activate various MMP precursors, gelatinase, and collagenase to degrade the extracellular matrix of cartilage and bone [30], and its increasing level is positively correlated with the degree of cartilage destruction [31, 32]. In our study, LIPUS effectively reduced MMP-3 expression levels in damaged condylar cartilage caused by CSD treatment. Beginning from 14 days, the effect of the LIPUS intervention was more definitive, and similarly, 45 mW/cm^2^ was the most effective intensity. Furthermore, this study found a negative correlation between COL-2 and MMP-3 expressions, suggesting that COL-2 degradation is closely associated with the function of MMP-3. However, how LIPUS affects MMP-3 expression is still unclear, and whether the MMP family is the most critical therapeutic target of LIPUS treatment requires further validation.

In summary, by combining the morphological results of condylar cartilage with immunohistochemistry analysis and molecular studies, we found that pathological changes in rat condylar cartilage tissue were significantly relieved when the LIPUS intervention lasted for 2 weeks, and 45 mW/cm^2^ was the most effective intensity. The limitation of the present study is that potential pathological changes in the synovial tissue were not reported. Previous studies have shown that LIPUS can ameliorate synovial inflammation in a destabilization of the medial meniscus (DMM) mouse model by inhibiting the production of mature IL1B/IL-1*β* and reducing the number of infiltrating inflammatory cells [33]. In addition, LIPUS was also confirmed to decrease the number of Cox-2-positive cells in the synovium of knee joints in a rheumatoid arthritis mouse model [34]. However, the pathogenic effect of CSD on synovial tissue and the mechanism underlying LIPUS treatment of synovitis of the temporomandibular joint still need further exploration.

## 5. Conclusions

We concluded that LIPUS applied for at least 2 weeks at an intensity of 45 mW/cm^2^ is the optimal LIPUS treatment regimen for temporomandibular joint injury in rats. The results provide a new reference for the selection of the strength and duration of LIPUS interventions and will serve as the foundation for our future studies on the molecular mechanism of LIPUS treatment for TMD.

## Figures and Tables

**Table 1:** Sequences of the primers used for RT-PCR.

Gene	Primer sequence (5′ to 3′)
PCNA	Forward: CGGCGTGAACCTACAGAGCATG
	Reverse: GCAGCGGTATGTGTCGAAGCC

COL-2	Forward: AAGAGCAAGGAGAAGAAG
	Reverse: TTACAGTGGTAGGTGATG

MMP-3	Forward: TGGACCAGGGACCAATGGA
	Reverse: GGCCAAGTTCATGAGCAGCA

*β*-Actin	Forward: ATGTGGATCAGCAAGCAGGA
	Reverse: GGTGTAAAACGCAGCTCAGTAA

## Data Availability

The data used to support the findings of this study are available from the corresponding author upon request.

## References

[1] Durham J., Newton-John T. R. O., Zakrzewska J. M. (2015). Temporomandibular disorders. *British Medical Journal*.

[2] Murphy M. K., MacBarb R. F., Wong M. E., Athanasiou K. A. (2013). Temporomandibular disorders: a review of etiology, clinical management, and tissue engineering strategies. *International Journal of Oral and Maxillofacial Implants*.

[3] Tanaka E., Detamore M. S., Mercuri L. G. (2008). Degenerative disorders of the temporomandibular joint: etiology, diagnosis, and treatment. *Journal of Dental Research*.

[4] Gauer R. L., Semidey M. J. (2015). Diagnosis and treatment of temporomandibular disorders. *American Family Physician*.

[5] Randhawa K., Bohay R., Côté P. (2016). The effectiveness of noninvasive interventions for temporomandibular disorders: a systematic review by the Ontario protocol for traffic injury management (OPTIMa) collaboration. *Clinical Journal of Pain*.

[6] Poolman R. W., Agoritsas T., Siemieniuk R. A. C. (2017). Low intensity pulsed ultrasound (LIPUS) for bone healing: a clinical practice guideline. *British Medical Journal*.

[7] Buarque de Gusmão C. V., Batista N. A., Vidotto Lemes V. T. (2019). Effect of low-intensity pulsed ultrasound stimulation, extracorporeal shockwaves and radial pressure waves on Akt, BMP-2, ERK-2, FAK and TGF-*β*1 during bone healing in rat tibial defects. *Ultrasound in Medicine and Biology*.

[8] Lou S., Lv H., Li Z., Zhang L., Tang P. (2017). The effects of low-intensity pulsed ultrasound on fresh fracture: a meta-analysis. *Medicine*.

[9] Xia B., Chen G., Zou Y., Yang L., Pan J., Lv Y. (2019). Low-intensity pulsed ultrasound combination with induced pluripotent stem cells-derived neural crest stem cells and growth differentiation factor 5 promotes sciatic nerve regeneration and functional recovery. *Journal of Tissue Engineering and Regenerative Medicine*.

[10] Kösters A. K., Ganse B., Gueorguiev B. (2017). Effects of low-intensity pulsed ultrasound on soft tissue micro-circulation in the foot. *International Orthopaedics*.

[11] Nishida T., Kubota S., Aoyama E., Yamanaka N., Lyons K. M., Takigawa M. (2017). Low-intensity pulsed ultrasound (LIPUS) treatment of cultured chondrocytes stimulates production of CCN family protein 2 (CCN2), a protein involved in the regeneration of articular cartilage: mechanism underlying this stimulation. *Osteoarthritis and Cartilage*.

[12] Fujita M., Sato-Shigeta M., Mori H. (2019). Protective effects of low-intensity pulsed ultrasound on mandibular condylar cartilage exposed to mechanical overloading. *Ultrasound in Medicine and Biology*.

[13] Kaur H., Uludağ H., Dederich D. N., El-Bialy T. (2017). Effect of increasing low-intensity pulsed ultrasound and a functional appliance on the mandibular condyle in growing rats. *Journal of Ultrasound in Medicine*.

[14] Geng W., Wu G., Huang F. (2015). Sleep deprivation induces abnormal bone metabolism in temporomandibular joint. *International Journal of Clinical and Experimental Medicine*.

[15] Oyonarte R., Zárate M., Rodriguez F. (2009). Low-intensity pulsed ultrasound stimulation of condylar growth in rats. *The Angle Orthodontist*.

[16] Mankin H. J., Dorfman H., Lippiello L., Zarins A. (1971). Biochemical and metabolic abnormalities in articular cartilage from osteo-arthritic human Hips. *The Journal of Bone and Joint Surgery American Volume*.

[17] Liang C., Yang T., Wu G., Li J., Geng W. (2019). Therapeutic effect of low-intensity pulsed ultrasound on temporomandibular joint injury induced by chronic sleep deprivation in rats. *American Journal of Translational Research*.

[18] Ma C., Wu G., Wang Z. (2014). Effects of chronic sleep deprivation on the extracellular signal-regulated kinase pathway in the temporomandibular joint of rats. *PLoS ONE*.

[19] Licini F., Nojelli A., Segu M., Collesano V. (2009). Role of psychosocial factors in the etiology of temporomandibular disorders: relevance of a biaxial diagnosis. *Minerva Stomatologica*.

[20] Ding F., Wang J., Zhu G., Zhao H., Wu G., Chen L. (2017). Osteopontin stimulates matrix metalloproteinase expression through the nuclear factor-*κ*B signaling pathway in rat temporomandibular joint and condylar chondrocytes. *American Journal of Translational Research*.

[21] de Sá M. P., Zanoni J. N., de Salles C. L., de Souza F. D., Suga U. S., Terada R. S. (2013). Morphometric evaluation of condylar cartilage of growing rats in response to mandibular retractive forces. *Dental Press Journal of Orthodontics*.

[22] Wang X. D., Kou X. X., Mao J. J., Gan Y. H., Zhou Y. H. (2012). Sustained inflammation induces degeneration of the temporomandibular joint. *Journal of Dental Research*.

[23] Hui T., Zhou Y., Wang T. (2018). Activation of *β*-catenin signaling in aggrecan-expressing cells in temporomandibular joint causes osteoarthritis-like defects. *International Journal of Oral Science*.

[24] Zhang S., Teo K. Y. W., Chuah S. J., Lai R. C., Lim S. K., Toh W. S. (2019). MSC exosomes alleviate temporomandibular joint osteoarthritis by attenuating inflammation and restoring matrix homeostasis. *Biomaterials*.

[25] Schönenberger F., Deutzmann A., Ferrando-May E., Merhof D. (2015). Discrimination of cell cycle phases in PCNA-immunolabeled cells. *BMC Bioinformatics*.

[26] Nazempour A., Van Wie B. J. (2016). Chondrocytes, mesenchymal stem cells, and their combination in articular cartilage regenerative medicine. *Annals of Biomedical Engineering*.

[27] Crossman J., Alzaheri N., Abdallah M. N. (2019). Low intensity pulsed ultrasound increases mandibular height and Col-II and VEGF expression in arthritic mice. *Archives of Oral Biology*.

[28] Simental-Mendía M., Lara-Arias J., Álvarez-Lozano E. (2015). Cotransfected human chondrocytes: over-expression of IGF-I and SOX9 enhances the synthesis of cartilage matrix components collagen-II and glycosaminoglycans. *Brazilian Journal of Medical and Biological Research*.

[29] Fukui T., Tenborg E., Yik J. H., Haudenschild D. R. (2015). In-vitro and in-vivo imaging of MMP activity in cartilage and joint injury. *Biochemical and Biophysical Research Communications*.

[30] Yu J., Mursu E., Typpö M. (2019). MMP-3 and MMP-8 in rat mandibular condylar cartilage associated with dietary loading, estrogen level, and aging. *Archives of Oral Biology*.

[31] Sun S., Bay-Jensen A. C., Karsdal M. A. (2014). The active form of MMP-3 is a marker of synovial inflammation and cartilage turnover in inflammatory joint diseases. *BMC Musculoskeletal Disorders*.

[32] Leong D. J., Gu X. I., Li Y. (2010). Matrix metalloproteinase-3 in articular cartilage is upregulated by joint immobilization and suppressed by passive joint motion. *Matrix Biology*.

[33] Zhang B., Chen H., Ouyang J. (2019). SQSTM1-dependent autophagic degradation of PKM2 inhibits the production of mature IL1B/IL-1*β* and contributes to LIPUS-mediated anti-inflammatory effect. *Autophagy*.

[34] Nakamura T., Fujihara S., Yamamoto-Nagata K., Katsura T., Inubushi T., Tanaka E. (2011). Low-intensity pulsed ultrasound reduces the inflammatory activity of synovitis. *Annals of Biomedical Engineering*.

